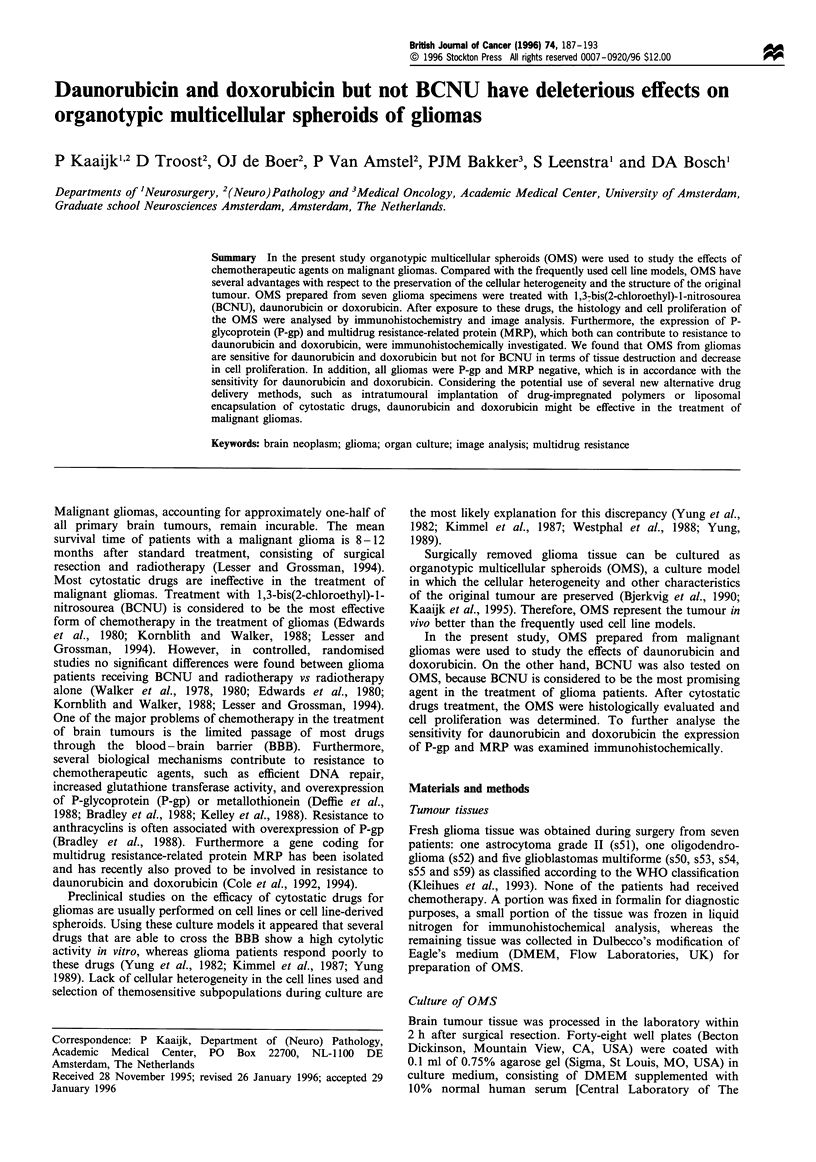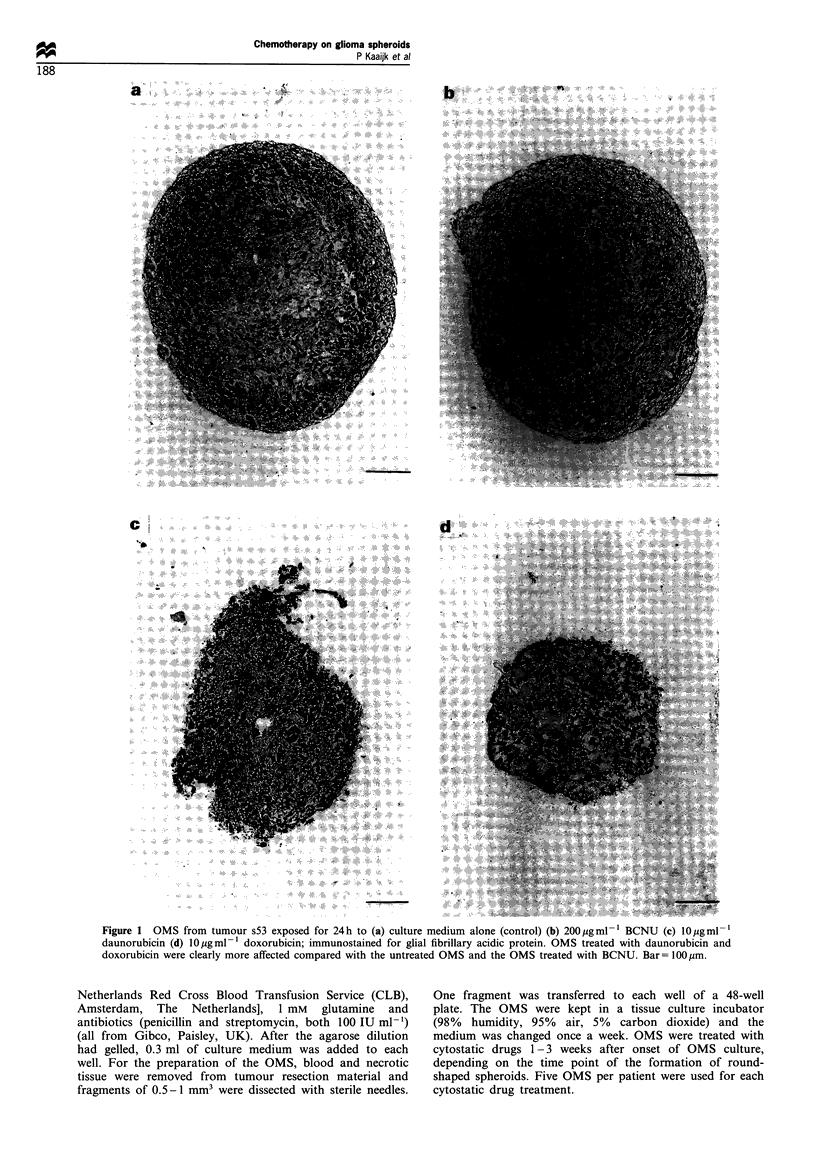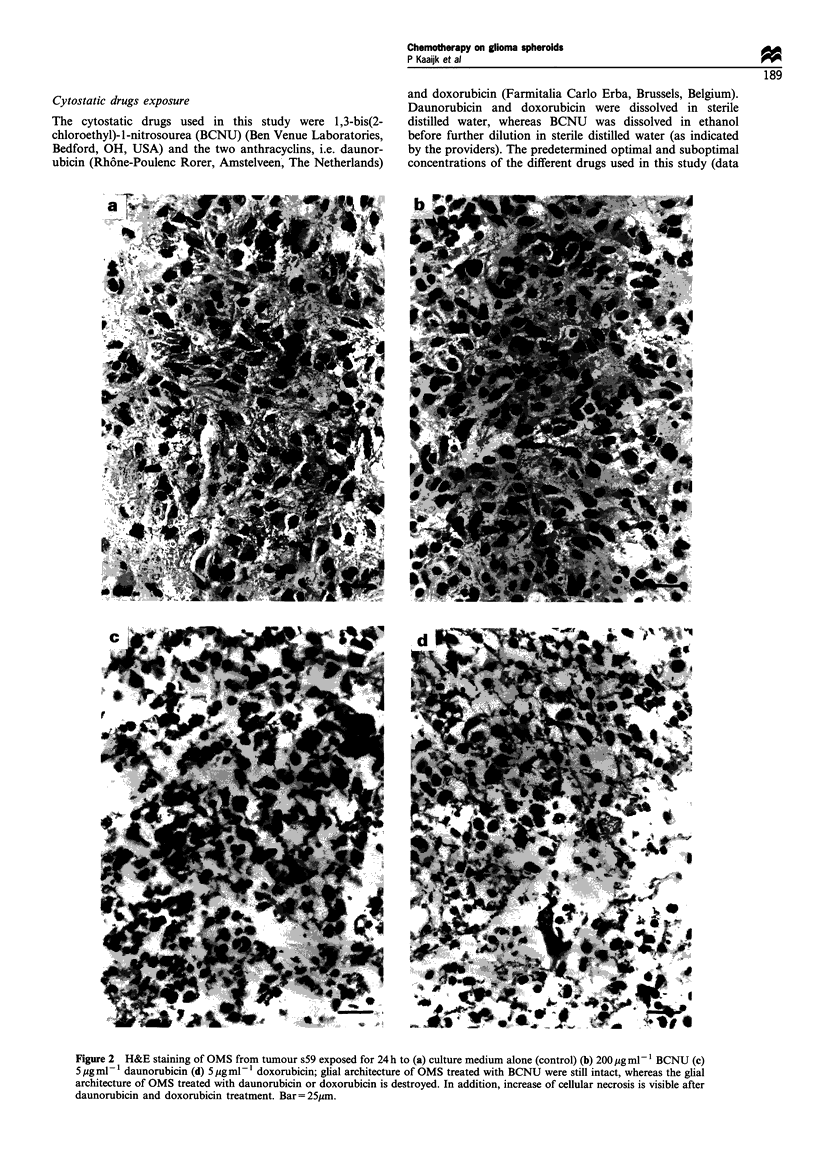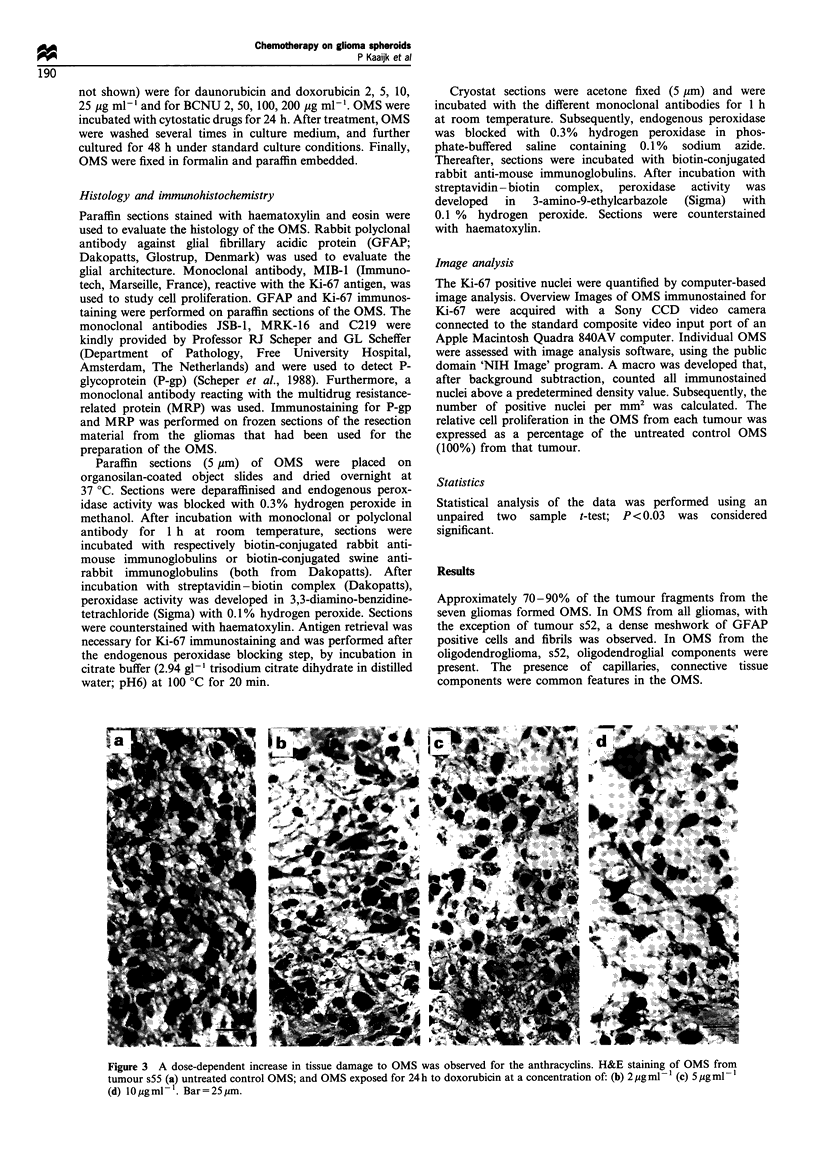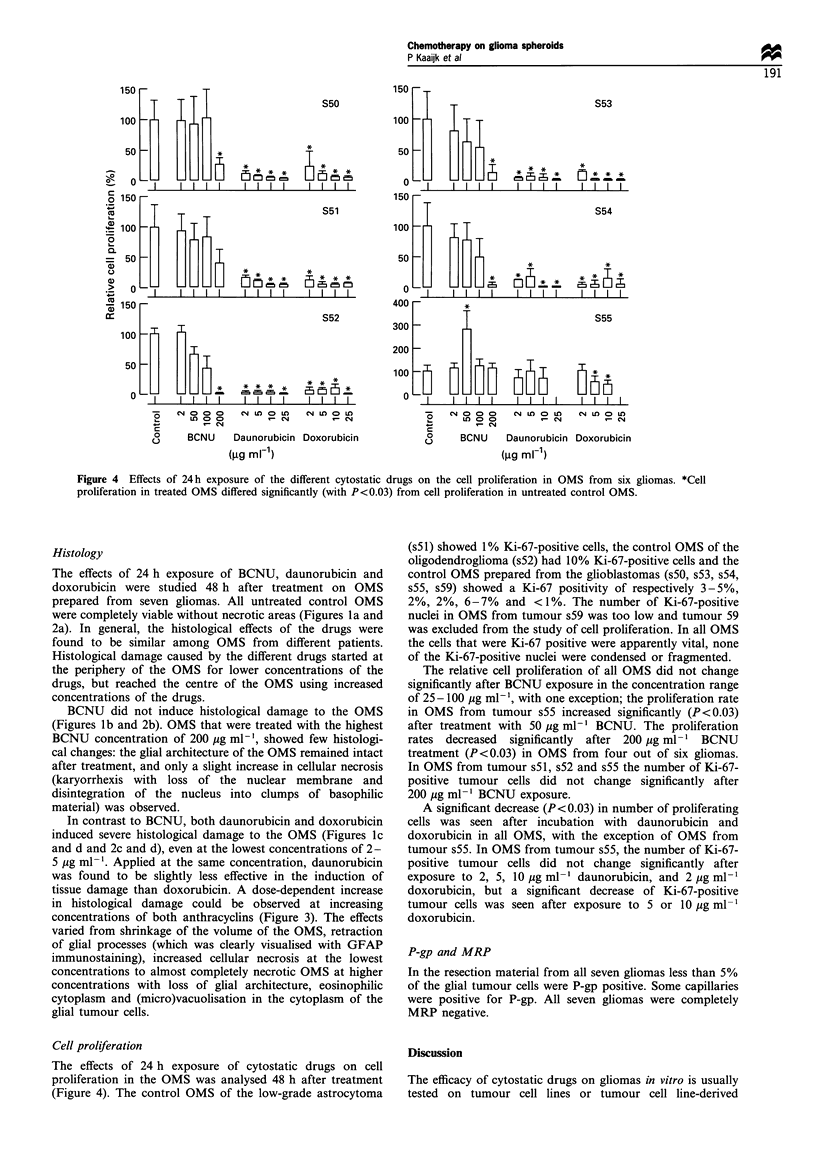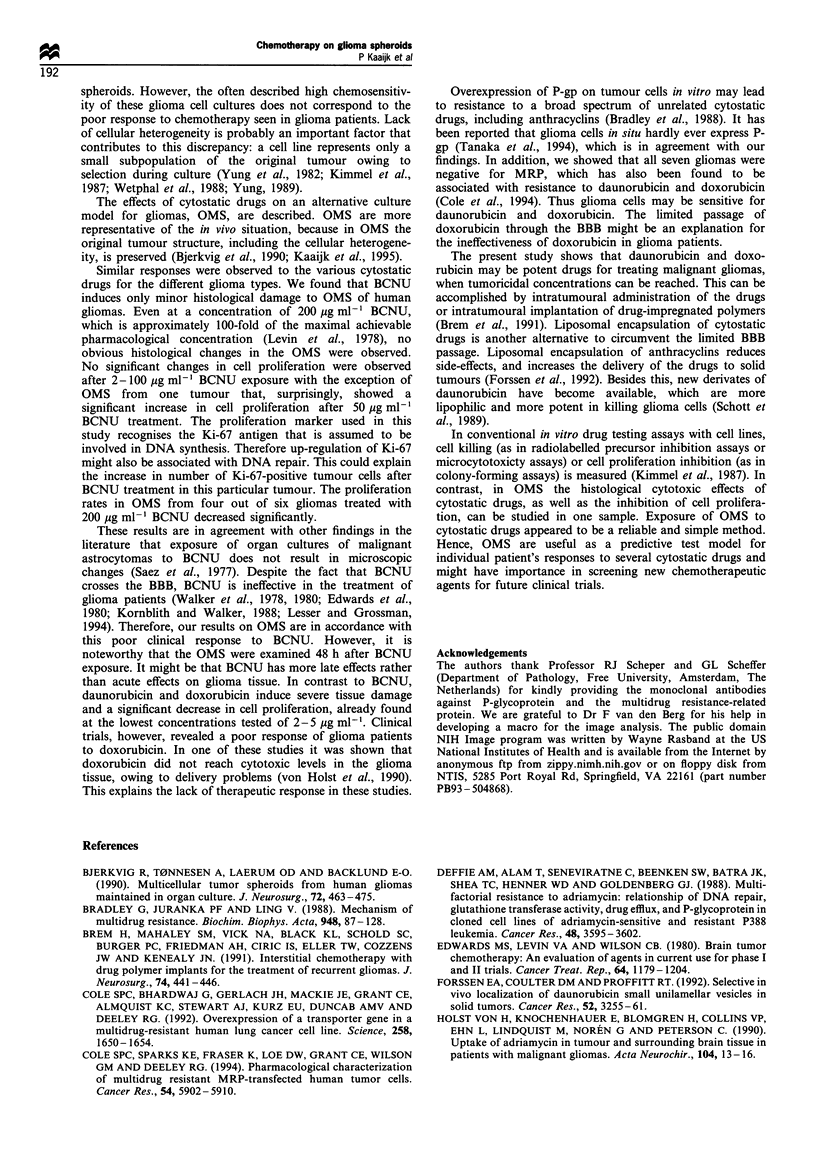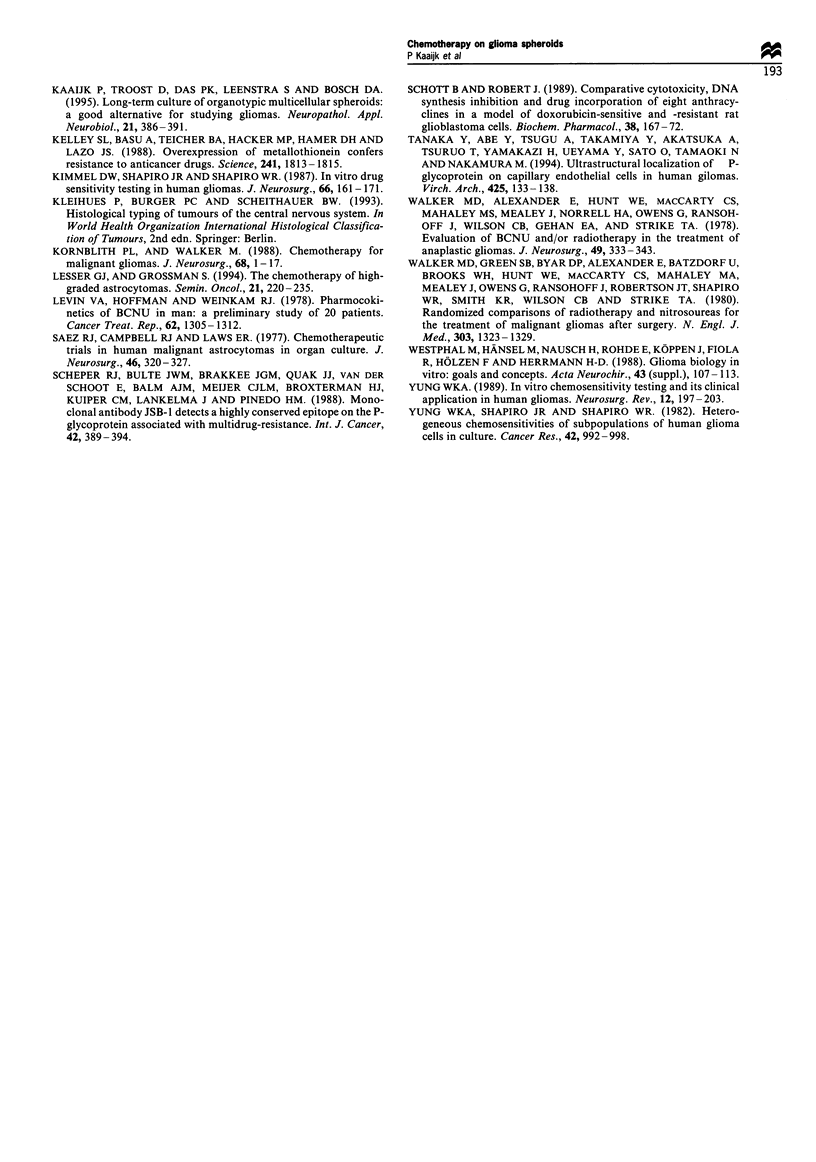# Daunorubicin and doxorubicin but not BCNU have deleterious effects on organotypic multicellular spheroids of gliomas.

**DOI:** 10.1038/bjc.1996.336

**Published:** 1996-07

**Authors:** P. Kaaijk, D. Troost, O. J. de Boer, P. Van Amstel, P. J. Bakker, S. Leenstra, D. A. Bosch

**Affiliations:** Department of Neurosurgery, University of Amsterdam, Graduate school Neurosciences Amsterdam, The Netherlands.

## Abstract

**Images:**


British Journal of Cancer (1996) 74, 187-193
© 1996 Stockton Press All rights reserved 0007-0920/96 $12.00

### Daunorubicin and doxorubicin but not BCNU have deleterious effects on organotypic multicellular spheroids of gliomas

P Kaaijk[1,2] D Troost[2], OJ de Boer[2], P Van Amstel[2], PJM Bakker[3], S Leenstra[1] and DA Bosch[1]

*Departments of [1]Neurosurgery, [2](Neuro)Pathology and [3]Medical Oncology, Academic Medical Center, University of Amsterdam, Graduate school Neurosciences Amsterdam, Amsterdam, The Netherlands.*

**Summary** In the present study organotypic multicellular spheroids (OMS) were used to study the effects of chemotherapeutic agents on malignant gliomas. Compared with the frequently used cell line models, OMS have several advantages with respect to the preservation of the cellular heterogeneity and the structure of the original tumour. OMS prepared from seven glioma specimens were treated with 1,3-bis(2-chloroethyl)-1-nitrosourea (BCNU), daunorubicin or doxorubicin. After exposure to these drugs, the histology and cell proliferation of the OMS were analysed by immunohistochemistry and image analysis. Furthermore, the expression of P-glycoprotein (P-gp) and multidrug resistance-related protein (MRP), which both can contribute to resistance to daunorubicin and doxorubicin, were immunohistochemically investigated. We found that OMS from gliomas are sensitive for daunorubicin and doxorubicin but not for BCNU in terms of tissue destruction and decrease in cell proliferation. In addition, all gliomas were P-gp and MRP negative, which is in accordance with the sensitivity for daunorubicin and doxorubicin. Considering the potential use of several new alternative drug delivery methods, such as intratumoural implantation of drug-impregnated polymers or liposomal encapsulation of cytostatic drugs, daunorubicin and doxorubicin might be effective in the treatment of malignant gliomas.

**Keywords:** brain neoplasm; glioma; organ culture; image analysis; multidrug resistance

Malignant gliomas, accounting for approximately one-half of all primary brain tumours, remain incurable. The mean survival time of patients with a malignant glioma is 8-12 months after standard treatment, consisting of surgical resection and radiotherapy (Lesser and Grossman, 1994). Most cytostatic drugs are ineffective in the treatment of malignant gliomas. Treatment with 1,3-bis(2-chloroethyl)-1-nitrosourea (BCNU) is considered to be the most effective form of chemotherapy in the treatment of gliomas (Edwards *et al.*, 1980; Kornblith and Walker, 1988; Lesser and Grossman, 1994). However, in controlled, randomised studies no significant differences were found between glioma patients receiving BCNU and radiotherapy *vs* radiotherapy alone (Walker *et al.*, 1978, 1980; Edwards *et al.*, 1980; Kornblith and Walker, 1988; Lesser and Grossman, 1994). One of the major problems of chemotherapy in the treatment of brain tumours is the limited passage of most drugs through the blood-brain barrier (BBB). Furthermore, several biological mechanisms contribute to resistance to chemotherapeutic agents, such as efficient DNA repair, increased glutathione transferase activity, and overexpression of P-glycoprotein (P-gp) or metallothionein (Deffie *et al.*, 1988; Bradley *et al.*, 1988; Kelley *et al.*, 1988). Resistance to anthracyclins is often associated with overexpression of P-gp (Bradley *et al.*, 1988). Furthermore a gene coding for multidrug resistance-related protein MRP has been isolated and has recently also proved to be involved in resistance to daunorubicin and doxorubicin (Cole *et al.*, 1992, 1994).

Preclinical studies on the efficacy of cytostatic drugs for gliomas are usually performed on cell lines or cell line-derived spheroids. Using these culture models it appeared that several drugs that are able to cross the BBB show a high cytolytic activity *in vitro*, whereas glioma patients respond poorly to these drugs (Yung *et al.*, 1982; Kimmel *et al.*, 1987; Yung 1989). Lack of cellular heterogeneity in the cell lines used and selection of themosensitive subpopulations during culture are the most likely explanation for this discrepancy (Yung *et al.*, 1982; Kimmel *et al.*, 1987; Westphal *et al.*, 1988; Yung, 1989).

Surgically removed glioma tissue can be cultured as organotypic multicellular spheroids (OMS), a culture model in which the cellular heterogeneity and other characteristics of the original tumour are preserved (Bjerkvig *et al.*, 1990; Kaaijk *et al.*, 1995). Therefore, OMS represent the tumour *in vivo* better than the frequently used cell line models.

In the present study, OMS prepared from malignant gliomas were used to study the effects of daunorubicin and doxorubicin. On the other hand, BCNU was also tested on OMS, because BCNU is considered to be the most promising agent in the treatment of glioma patients. After cytostatic drugs treatment, the OMS were histologically evaluated and cell proliferation was determined. To further analyse the sensitivity for daunorubicin and doxorubicin the expression of P-gp and MRP was examined immunohistochemically.

### Materials and methods

### *Tumour tissues*

Fresh glioma tissue was obtained during surgery from seven patients: one astrocytoma grade II (s51), one oligodendroglioma (s52) and five glioblastomas multiforme (s50, s53, s54, s55 and s59) as classified according to the WHO classification (Kleihues *et al.*, 1993). None of the patients had received chemotherapy. A portion was fixed in formalin for diagnostic purposes, a small portion of the tissue was frozen in liquid nitrogen for immunohistochemical analysis, whereas the remaining tissue was collected in Dulbecco's modification of Eagle's medium (DMEM, Flow Laboratories, UK) for preparation of OMS.

### *Culture of OMS*

Brain tumour tissue was processed in the laboratory within 2 h after surgical resection. Forty-eight well plates (Becton Dickinson, Mountain View, CA, USA) were coated with 0.1 ml of 0.75% agarose gel (Sigma, St Louis, MO, USA) in culture medium, consisting of DMEM supplemented with 10% normal human serum [Central Laboratory of The

Correspondence: P Kaaijk, Department of (Neuro) Pathology, Academic Medical Center, PO Box 22700, NL-1100 DE Amsterdam, The Netherlands
Received 28 November 1995; revised 26 January 1996; accepted 29 January 1996

**Figure 1** OMS from tumour s53 exposed for 24 h to (**a**) culture medium alone (control) (**b**) 200 $\mu$g ml$^{-1}$ BCNU (**c**) 10 $\mu$g ml$^{-1}$ daunorubicin (**d**) 10 $\mu$g ml$^{-1}$ doxorubicin; immunostained for glial fibrillary acidic protein. OMS treated with daunorubicin and doxorubicin were clearly more affected compared with the untreated OMS and the OMS treated with BCNU. Bar = 100 $\mu$m.

Netherlands Red Cross Blood Transfusion Service (CLB), Amsterdam, The Netherlands], 1 mM glutamine and antibiotics (penicillin and streptomycin, both 100 IU ml$^{-1}$) (all from Gibco, Paisley, UK). After the agarose dilution had gelled, 0.3 ml of culture medium was added to each well. For the preparation of the OMS, blood and necrotic tissue were removed from tumour resection material and fragments of 0.5–1 mm$^3$ were dissected with sterile needles. One fragment was transferred to each well of a 48-well plate. The OMS were kept in a tissue culture incubator (98% humidity, 95% air, 5% carbon dioxide) and the medium was changed once a week. OMS were treated with cytostatic drugs 1–3 weeks after onset of OMS culture, depending on the time point of the formation of round-shaped spheroids. Five OMS per patient were used for each cytostatic drug treatment.

*Cytostatic drugs exposure*

The cytostatic drugs used in this study were 1,3-bis(2-chloroethyl)-1-nitrosourea (BCNU) (Ben Venue Laboratories, Bedford, OH, USA) and the two anthracyclins, i.e. daunorubicin (Rhône-Poulenc Rorer, Amstelveen, The Netherlands) and doxorubicin (Farmitalia Carlo Erba, Brussels, Belgium). Daunorubicin and doxorubicin were dissolved in sterile distilled water, whereas BCNU was dissolved in ethanol before further dilution in sterile distilled water (as indicated by the providers). The predetermined optimal and suboptimal concentrations of the different drugs used in this study (data

**Figure 2** H&E staining of OMS from tumour s59 exposed for 24 h to (**a**) culture medium alone (control) (**b**) 200 $\mu g\,ml^{-1}$ BCNU (**c**) 5 $\mu g\,ml^{-1}$ daunorubicin (**d**) 5 $\mu g\,ml^{-1}$ doxorubicin; glial architecture of OMS treated with BCNU were still intact, whereas the glial architecture of OMS treated with daunorubicin or doxorubicin is destroyed. In addition, increase of cellular necrosis is visible after daunorubicin and doxorubicin treatment. Bar = 25 $\mu m$.

not shown) were for daunorubicin and doxorubicin 2, 5, 10, 25 $\mu$g ml$^{-1}$ and for BCNU 2, 50, 100, 200 $\mu$g ml$^{-1}$. OMS were incubated with cytostatic drugs for 24 h. After treatment, OMS were washed several times in culture medium, and further cultured for 48 h under standard culture conditions. Finally, OMS were fixed in formalin and paraffin embedded.

### *Histology and immunohistochemistry*

Paraffin sections stained with haematoxylin and eosin were used to evaluate the histology of the OMS. Rabbit polyclonal antibody against glial fibrillary acidic protein (GFAP; Dakopatts, Glostrup, Denmark) was used to evaluate the glial architecture. Monoclonal antibody, MIB-1 (Immunotech, Marseille, France), reactive with the Ki-67 antigen, was used to study cell proliferation. GFAP and Ki-67 immunostaining were performed on paraffin sections of the OMS. The monoclonal antibodies JSB-1, MRK-16 and C219 were kindly provided by Professor RJ Scheper and GL Scheffer (Department of Pathology, Free University Hospital, Amsterdam, The Netherlands) and were used to detect P-glycoprotein (P-gp) (Scheper *et al.*, 1988). Furthermore, a monoclonal antibody reacting with the multidrug resistance-related protein (MRP) was used. Immunostaining for P-gp and MRP was performed on frozen sections of the resection material from the gliomas that had been used for the preparation of the OMS.

Paraffin sections (5 $\mu$m) of OMS were placed on organosilan-coated object slides and dried overnight at 37 °C. Sections were deparaffinised and endogenous peroxidase activity was blocked with 0.3% hydrogen peroxide in methanol. After incubation with monoclonal or polyclonal antibody for 1 h at room temperature, sections were incubated with respectively biotin-conjugated rabbit anti-mouse immunoglobulins or biotin-conjugated swine anti-rabbit immunoglobulins (both from Dakopatts). After incubation with streptavidin–biotin complex (Dakopatts), peroxidase activity was developed in 3,3-diamino-benzidine-tetrachloride (Sigma) with 0.1% hydrogen peroxide. Sections were counterstained with haematoxylin. Antigen retrieval was necessary for Ki-67 immunostaining and was performed after the endogenous peroxidase blocking step, by incubation in citrate buffer (2.94 gl$^{-1}$ trisodium citrate dihydrate in distilled water; pH6) at 100 °C for 20 min.

Cryostat sections were acetone fixed (5 $\mu$m) and were incubated with the different monoclonal antibodies for 1 h at room temperature. Subsequently, endogenous peroxidase was blocked with 0.3% hydrogen peroxidase in phosphate-buffered saline containing 0.1% sodium azide. Thereafter, sections were incubated with biotin-conjugated rabbit anti-mouse immunoglobulins. After incubation with streptavidin–biotin complex, peroxidase activity was developed in 3-amino-9-ethylcarbazole (Sigma) with 0.1 % hydrogen peroxide. Sections were counterstained with haematoxylin.

### *Image analysis*

The Ki-67 positive nuclei were quantified by computer-based image analysis. Overview Images of OMS immunostained for Ki-67 were acquired with a Sony CCD video camera connected to the standard composite video input port of an Apple Macintosh Quadra 840AV computer. Individual OMS were assessed with image analysis software, using the public domain 'NIH Image' program. A macro was developed that, after background subtraction, counted all immunostained nuclei above a predetermined density value. Subsequently, the number of positive nuclei per mm$^2$ was calculated. The relative cell proliferation in the OMS from each tumour was expressed as a percentage of the untreated control OMS (100%) from that tumour.

### *Statistics*

Statistical analysis of the data was performed using an unpaired two sample $t$-test; $P<0.03$ was considered significant.

### Results

Approximately 70–90% of the tumour fragments from the seven gliomas formed OMS. In OMS from all gliomas, with the exception of tumour s52, a dense meshwork of GFAP positive cells and fibrils was observed. In OMS from the oligodendroglioma, s52, oligodendroglial components were present. The presence of capillaries, connective tissue components were common features in the OMS.

**Figure 3** A dose-dependent increase in tissue damage to OMS was observed for the anthracyclins. H&E staining of OMS from tumour s55 (**a**) untreated control OMS; and OMS exposed for 24 h to doxorubicin at a concentration of: (**b**) 2 $\mu$g ml$^{-1}$ (**c**) 5 $\mu$g ml$^{-1}$ (**d**) 10 $\mu$g ml$^{-1}$. Bar = 25 $\mu$m.

**Figure 4** Effects of 24 h exposure of the different cytostatic drugs on the cell proliferation in OMS from six gliomas. *Cell proliferation in treated OMS differed significantly (with $P<0.03$) from cell proliferation in untreated control OMS.

*Histology*

The effects of 24 h exposure of BCNU, daunorubicin and doxorubicin were studied 48 h after treatment on OMS prepared from seven gliomas. All untreated control OMS were completely viable without necrotic areas (Figures 1a and 2a). In general, the histological effects of the drugs were found to be similar among OMS from different patients. Histological damage caused by the different drugs started at the periphery of the OMS for lower concentrations of the drugs, but reached the centre of the OMS using increased concentrations of the drugs.

BCNU did not induce histological damage to the OMS (Figures 1b and 2b). OMS that were treated with the highest BCNU concentration of 200 $\mu g\ ml^{-1}$, showed few histological changes: the glial architecture of the OMS remained intact after treatment, and only a slight increase in cellular necrosis (karyorrhexis with loss of the nuclear membrane and disintegration of the nucleus into clumps of basophilic material) was observed.

In contrast to BCNU, both daunorubicin and doxorubicin induced severe histological damage to the OMS (Figures 1c and d and 2c and d), even at the lowest concentrations of 2–5 $\mu g\ ml^{-1}$. Applied at the same concentration, daunorubicin was found to be slightly less effective in the induction of tissue damage than doxorubicin. A dose-dependent increase in histological damage could be observed at increasing concentrations of both anthracyclins (Figure 3). The effects varied from shrinkage of the volume of the OMS, retraction of glial processes (which was clearly visualised with GFAP immunostaining), increased cellular necrosis at the lowest concentrations to almost completely necrotic OMS at higher concentrations with loss of glial architecture, eosinophilic cytoplasm and (micro)vacuolisation in the cytoplasm of the glial tumour cells.

*Cell proliferation*

The effects of 24 h exposure of cytostatic drugs on cell proliferation in the OMS was analysed 48 h after treatment (Figure 4). The control OMS of the low-grade astrocytoma (s51) showed 1% Ki-67-positive cells, the control OMS of the oligodendroglioma (s52) had 10% Ki-67-positive cells and the control OMS prepared from the glioblastomas (s50, s53, s54, s55, s59) showed a Ki-67 positivity of respectively 3–5%, 2%, 2%, 6–7% and <1%. The number of Ki-67-positive nuclei in OMS from tumour s59 was too low and tumour 59 was excluded from the study of cell proliferation. In all OMS the cells that were Ki-67 positive were apparently vital, none of the Ki-67-positive nuclei were condensed or fragmented.

The relative cell proliferation of all OMS did not change significantly after BCNU exposure in the concentration range of 25–100 $\mu g\ ml^{-1}$, with one exception; the proliferation rate in OMS from tumour s55 increased significantly ($P<0.03$) after treatment with 50 $\mu g\ ml^{-1}$ BCNU. The proliferation rates decreased significantly after 200 $\mu g\ ml^{-1}$ BCNU treatment ($P<0.03$) in OMS from four out of six gliomas. In OMS from tumour s51, s52 and s55 the number of Ki-67-positive tumour cells did not change significantly after 200 $\mu g\ ml^{-1}$ BCNU exposure.

A significant decrease ($P<0.03$) in number of proliferating cells was seen after incubation with daunorubicin and doxorubicin in all OMS, with the exception of OMS from tumour s55. In OMS from tumour s55, the number of Ki-67-positive tumour cells did not change significantly after exposure to 2, 5, 10 $\mu g\ ml^{-1}$ daunorubicin, and 2 $\mu g\ ml^{-1}$ doxorubicin, but a significant decrease of Ki-67-positive tumour cells was seen after exposure to 5 or 10 $\mu g\ ml^{-1}$ doxorubicin.

*P-gp and MRP*

In the resection material from all seven gliomas less than 5% of the glial tumour cells were P-gp positive. Some capillaries were positive for P-gp. All seven gliomas were completely MRP negative.

### Discussion

The efficacy of cytostatic drugs on gliomas *in vitro* is usually tested on tumour cell lines or tumour cell line-derived spheroids. However, the often described high chemosensitivity of these glioma cell cultures does not correspond to the poor response to chemotherapy seen in glioma patients. Lack of cellular heterogeneity is probably an important factor that contributes to this discrepancy: a cell line represents only a small subpopulation of the original tumour owing to selection during culture (Yung *et al.*, 1982; Kimmel *et al.*, 1987; Wetphal *et al.*, 1988; Yung, 1989).

The effects of cytostatic drugs on an alternative culture model for gliomas, OMS, are described. OMS are more representative of the *in vivo* situation, because in OMS the original tumour structure, including the cellular heterogeneity, is preserved (Bjerkvig *et al.*, 1990; Kaaijk *et al.*, 1995).

Similar responses were observed to the various cytostatic drugs for the different glioma types. We found that BCNU induces only minor histological damage to OMS of human gliomas. Even at a concentration of 200 $\mu g\ ml^{-1}$ BCNU, which is approximately 100-fold of the maximal achievable pharmacological concentration (Levin *et al.*, 1978), no obvious histological changes in the OMS were observed. No significant changes in cell proliferation were observed after 2–100 $\mu g\ ml^{-1}$ BCNU exposure with the exception of OMS from one tumour that, surprisingly, showed a significant increase in cell proliferation after 50 $\mu g\ ml^{-1}$ BCNU treatment. The proliferation marker used in this study recognises the Ki-67 antigen that is assumed to be involved in DNA synthesis. Therefore up-regulation of Ki-67 might also be associated with DNA repair. This could explain the increase in number of Ki-67-positive tumour cells after BCNU treatment in this particular tumour. The proliferation rates in OMS from four out of six gliomas treated with 200 $\mu g\ ml^{-1}$ BCNU decreased significantly.

These results are in agreement with other findings in the literature that exposure of organ cultures of malignant astrocytomas to BCNU does not result in microscopic changes (Saez *et al.*, 1977). Despite the fact that BCNU crosses the BBB, BCNU is ineffective in the treatment of glioma patients (Walker *et al.*, 1978, 1980; Edwards *et al.*, 1980; Kornblith and Walker, 1988; Lesser and Grossman, 1994). Therefore, our results on OMS are in accordance with this poor clinical response to BCNU. However, it is noteworthy that the OMS were examined 48 h after BCNU exposure. It might be that BCNU has more late effects rather than acute effects on glioma tissue. In contrast to BCNU, daunorubicin and doxorubicin induce severe tissue damage and a significant decrease in cell proliferation, already found at the lowest concentrations tested of 2–5 $\mu g\ ml^{-1}$. Clinical trials, however, revealed a poor response of glioma patients to doxorubicin. In one of these studies it was shown that doxorubicin did not reach cytotoxic levels in the glioma tissue, owing to delivery problems (von Holst *et al.*, 1990). This explains the lack of therapeutic response in these studies.

Overexpression of P-gp on tumour cells *in vitro* may lead to resistance to a broad spectrum of unrelated cytostatic drugs, including anthracyclins (Bradley *et al.*, 1988). It has been reported that glioma cells *in situ* hardly ever express P-gp (Tanaka *et al.*, 1994), which is in agreement with our findings. In addition, we showed that all seven gliomas were negative for MRP, which has also been found to be associated with resistance to daunorubicin and doxorubicin (Cole *et al.*, 1994). Thus glioma cells may be sensitive for daunorubicin and doxorubicin. The limited passage of doxorubicin through the BBB might be an explanation for the ineffectiveness of doxorubicin in glioma patients.

The present study shows that daunorubicin and doxorubicin may be potent drugs for treating malignant gliomas, when tumoricidal concentrations can be reached. This can be accomplished by intratumoural administration of the drugs or intratumoural implantation of drug-impregnated polymers (Brem *et al.*, 1991). Liposomal encapsulation of cytostatic drugs is another alternative to circumvent the limited BBB passage. Liposomal encapsulation of anthracyclins reduces side-effects, and increases the delivery of the drugs to solid tumours (Forssen *et al.*, 1992). Besides this, new derivates of daunorubicin have become available, which are more lipophilic and more potent in killing glioma cells (Schott *et al.*, 1989).

In conventional *in vitro* drug testing assays with cell lines, cell killing (as in radiolabelled precursor inhibition assays or microcytotoxicty assays) or cell proliferation inhibition (as in colony-forming assays) is measured (Kimmel *et al.*, 1987). In contrast, in OMS the histological cytotoxic effects of cytostatic drugs, as well as the inhibition of cell proliferation, can be studied in one sample. Exposure of OMS to cytostatic drugs appeared to be a reliable and simple method. Hence, OMS are useful as a predictive test model for individual patient's responses to several cytostatic drugs and might have importance in screening new chemotherapeutic agents for future clinical trials.

**Acknowledgements**

The authors thank Professor RJ Scheper and GL Scheffer (Department of Pathology, Free University, Amsterdam, The Netherlands) for kindly providing the monoclonal antibodies against P-glycoprotein and the multidrug resistance-related protein. We are grateful to Dr F van den Berg for his help in developing a macro for the image analysis. The public domain NIH Image program was written by Wayne Rasband at the US National Institutes of Health and is available from the Internet by anonymous ftp from zippy.nimh.nih.gov or on floppy disk from NTIS, 5285 Port Royal Rd, Springfield, VA 22161 (part number PB93–504868).

### References

BJERKVIG R, TØNNESEN A, LAERUM OD AND BACKLUND E-O. (1990). Multicellular tumor spheroids from human gliomas maintained in organ culture. *J. Neurosurg.*, **72**, 463–475.

BRADLEY G, JURANKA PF AND LING V. (1988). Mechanism of multidrug resistance. *Biochim. Biophys. Acta*, **948**, 87–128.

BREM H, MAHALEY SM, VICK NA, BLACK KL, SCHOLD SC, BURGER PC, FRIEDMAN AH, CIRIC IS, ELLER TW, COZZENS JW AND KENEALY JN. (1991). Interstitial chemotherapy with drug polymer implants for the treatment of recurrent gliomas. *J. Neurosurg.*, **74**, 441–446.

COLE SPC, BHARDWAJ G, GERLACH JH, MACKIE JE, GRANT CE, ALMQUIST KC, STEWART AJ, KURZ EU, DUNCAB AMV AND DEELEY RG. (1992). Overexpression of a transporter gene in a multidrug-resistant human lung cancer cell line. *Science*, **258**, 1650–1654.

COLE SPC, SPARKS KE, FRASER K, LOE DW, GRANT CE, WILSON GM AND DEELEY RG. (1994). Pharmacological characterization of multidrug resistant MRP-transfected human tumor cells. *Cancer Res.*, **54**, 5902–5910.

DEFFIE AM, ALAM T, SENEVIRATNE C, BEENKEN SW, BATRA JK, SHEA TC, HENNER WD AND GOLDENBERG GJ. (1988). Multifactorial resistance to adriamycin: relationship of DNA repair, glutathione transferase activity, drug efflux, and P-glycoprotein in cloned cell lines of adriamycin-sensitive and resistant P388 leukemia. *Cancer Res.*, **48**, 3595–3602.

EDWARDS MS, LEVIN VA AND WILSON CB. (1980). Brain tumor chemotherapy: An evaluation of agents in current use for phase I and II trials. *Cancer Treat. Rep.*, **64**, 1179–1204.

FORSSEN EA, COULTER DM AND PROFFITT RT. (1992). Selective in vivo localization of daunorubicin small unilamellar vesicles in solid tumors. *Cancer Res.*, **52**, 3255–61.

HOLST VON H, KNOCHENHAUER E, BLOMGREN H, COLLINS VP, EHN L, LINDQUIST M, NORÉN G AND PETERSON C. (1990). Uptake of adriamycin in tumour and surrounding brain tissue in patients with malignant gliomas. *Acta Neurochir.*, **104**, 13–16.

KAAIJK P, TROOST D, DAS PK, LEENSTRA S AND BOSCH DA. (1995). Long-term culture of organotypic multicellular spheroids: a good alternative for studying gliomas. *Neuropathol. Appl. Neurobiol.*, **21,** 386–391.

KELLEY SL, BASU A, TEICHER BA, HACKER MP, HAMER DH AND LAZO JS. (1988). Overexpression of metallothionein confers resistance to anticancer drugs. *Science*, **241,** 1813–1815.

KIMMEL DW, SHAPIRO JR AND SHAPIRO WR. (1987). In vitro drug sensitivity testing in human gliomas. *J. Neurosurg.*, **66,** 161–171.

KLEIHUES P, BURGER PC AND SCHEITHAUER BW. (1993). Histological typing of tumours of the central nervous system. *In World Health Organization International Histological Classification of Tumours*, 2nd edn. Springer: Berlin.

KORNBLITH PL, AND WALKER M. (1988). Chemotherapy for malignant gliomas. *J. Neurosurg.*, **68,** 1–17.

LESSER GJ, AND GROSSMAN S. (1994). The chemotherapy of high-graded astrocytomas. *Semin. Oncol.*, **21,** 220–235.

LEVIN VA, HOFFMAN AND WEINKAM RJ. (1978). Pharmocokinetics of BCNU in man: a preliminary study of 20 patients. *Cancer Treat. Rep.*, **62,** 1305–1312.

SAEZ RJ, CAMPBELL RJ AND LAWS ER. (1977). Chemotherapeutic trials in human malignant astrocytomas in organ culture. *J. Neurosurg.*, **46,** 320–327.

SCHEPER RJ, BULTE JWM, BRAKKEE JGM, QUAK JJ, VAN DER SCHOOT E, BALM AJM, MEIJER CJLM, BROXTERMAN HJ, KUIPER CM, LANKELMA J AND PINEDO HM. (1988). Monoclonal antibody JSB-1 detects a highly conserved epitope on the P-glycoprotein associated with multidrug-resistance. *Int. J. Cancer*, **42,** 389–394.

SCHOTT B AND ROBERT J. (1989). Comparative cytotoxicity, DNA synthesis inhibition and drug incorporation of eight anthracyclines in a model of doxorubicin-sensitive and -resistant rat glioblastoma cells. *Biochem. Pharmacol.*, **38,** 167–72.

TANAKA Y, ABE Y, TSUGU A, TAKAMIYA Y, AKATSUKA A, TSURUO T, YAMAKAZI H, UEYAMA Y, SATO O, TAMAOKI N AND NAKAMURA M. (1994). Ultrastructural localization of P-glycoprotein on capillary endothelial cells in human gilomas. *Virch. Arch.*, **425,** 133–138.

WALKER MD, ALEXANDER E, HUNT WE, MACCARTY CS, MAHALEY MS, MEALEY J, NORRELL HA, OWENS G, RANSOHOFF J, WILSON CB, GEHAN EA, AND STRIKE TA. (1978). Evaluation of BCNU and/or radiotherapy in the treatment of anaplastic gliomas. *J. Neurosurg.*, **49,** 333–343.

WALKER MD, GREEN SB, BYAR DP, ALEXANDER E, BATZDORF U, BROOKS WH, HUNT WE, MACCARTY CS, MAHALEY MA, MEALEY J, OWENS G, RANSOHOFF J, ROBERTSON JT, SHAPIRO WR, SMITH KR, WILSON CB AND STRIKE TA. (1980). Randomized comparisons of radiotherapy and nitrosoureas for the treatment of malignant gliomas after surgery. *N. Engl. J. Med.*, **303,** 1323–1329.

WESTPHAL M, HÄNSEL M, NAUSCH H, ROHDE E, KÖPPEN J, FIOLA R, HÖLZEN F AND HERRMANN H-D. (1988). Glioma biology in vitro: goals and concepts. *Acta Neurochir.*, **43** (suppl.), 107–113.

YUNG WKA. (1989). In vitro chemosensitivity testing and its clinical application in human gliomas. *Neurosurg. Rev.*, **12,** 197–203.

YUNG WKA, SHAPIRO JR AND SHAPIRO WR. (1982). Heterogeneous chemosensitivities of subpopulations of human glioma cells in culture. *Cancer Res.*, **42,** 992–998.